# Electrospun Fibres with Hyaluronic Acid-Chitosan Nanoparticles Produced by a Portable Device

**DOI:** 10.3390/nano10102016

**Published:** 2020-10-13

**Authors:** Carla V. Fuenteslópez, Hua Ye

**Affiliations:** Department of Engineering Science, Institute of Biomedical Engineering, University of Oxford, Oxford OX3 7DQ, UK; carla.fuenteslopez@eng.ox.ac.uk

**Keywords:** nanoparticles, electrospinning, hyaluronic acid, chitosan, portable electrospinning device, polycaprolactone, gelatine

## Abstract

Electrospinning is a versatile technique to produce nano/microscale fibrous scaffolds for tissue engineering and drug delivery applications. This research aims to demonstrate that hyaluronic acid-chitosan (HA-CS) nanoparticles can be electrospun together with polycaprolactone (PCL) and gelatine (Ge) fibres using a portable device to create scaffolds for tissue repair. A range of polymer solutions of PCL-gelatine at different weight/volume concentrations and ratios were electrospun and characterised. Fibre–cell interaction (F11 cells) was evaluated based on cell viability and proliferation and, from here, a few polymer blends were electrospun into random or aligned fibre arrangements. HA-CS nanoparticles were synthesised, characterised, and used to functionalise electrospun fibres (8% *w*/*v* at 70 PCL:30 Ge), which were chosen based on cell viability. Different concentrations of HA-CS nanoparticles were tested to determine cytotoxicity. A single dosage (1 × 10^−2^ mg/mL) was associated with higher cell proliferation compared with the cell-only control. This nanoparticle concentration was embedded into the electrospun fibres as either surface modification or blend. Fibres with blended NPs delivered a higher cell viability than unmodified fibres, while NP-coated fibres resulted in a higher cell proliferation (72 h) than the NP-blended ones. These biocompatible scaffolds allow cell attachment, maintain fibre arrangement, promote directional growth and yield higher cell viability.

## 1. Introduction

Tissues such as nerves, cartilage and bone have a limited capacity for regeneration, especially when the damage is extensive [1]. In order to address this challenge, tissue engineering (TE) combines scaffolds (i.e., materials), signalling molecules and cells to create constructs that support cell growth, replace damaged biological tissues and guide tissue regeneration [2].

Electrospun fibres have been widely used as scaffolds for TE, as they are able to partially mimic the structure and spatial topographies of the natural extracellular matrix (ECM) [3,4], and therefore improve cell adhesion, proliferation and differentiation [5], as well as reducing implant rejection [6]. Moreover, the structures produced by electrospinning yield excellent mechanical properties [7] and offer unique ones such as a high surface-area-to-volume ration and interfibrous porosity [5,6,7].

One of the main advantages of electrospinning is the simplicity of the device which, coupled with the technique being easily accessible, highly effective, and an efficient method for fibre fabrication [8], makes it a very attractive technique for scaffold fabrication. There are different ways to perform electrospinning, including needle-based (uses a hollow needle as the spinneret) and needleless (opts for the fabrication of fibres directly from an open liquid surface) [9]. Typically, an apparatus comprises (1) a high-voltage power supply, (2) a tube with small diameter that is connected to a syringe pump (i.e., a dispenser) and (3) a grounded collector [5,10]. Needle-based electrospinning is easy to set up, can use a wide variety of materials to be easily processed, offers tightly controlled flow rate and minimises solution waste [9].

Furthermore, electrospinning is a very versatile technique that can achieve, among other things, different fibre morphologies and orientation thanks to the range of spinnerets and collectors available [7]. Spinnerets for needle-based electrospinning include porous hollow tubes, coaxial nozzles and multi-nozzles [9]. In terms of the collectors available, rotating drums, rings, conveyors, parallel electrodes, and exterior frames [9], to name a few, make it possible to produce aligned fibres; while patterned collectors are used for more complex geometries [11].

Moreover, devices can be classified into bench-top and portable. This last category includes hand-held spinnerets, battery-powered, and generator-powered devices [7]. While benchtop devices are able to produce high quality and reproducible samples, they lack flexibility. This limits their utility in many key applications, such as direct deposition of fibres and the creation of a range of fibre arrangements. Portable electrospinning devices aim to address these challenges. A brief comparison of benchtop and portable apparatus is presented in Table 1.

Portable electrospinners are particularly useful for direct deposition of fibres onto the target site [12]. In situ fibre deposition onto wound sites [7], especially thermal, traumatic and chronic wounds [13], allows it to be tailored to individual patients. This makes it possible to manage wound sites quickly, promoting healing [14]. Moreover, the small sized, lightweight and battery/generator-powered devices allow for these to be operated in most locations, including emergency medical transport [13], hospitals, clinics and patients’ homes, but also remote areas including humanitarian and low-resource settings.

The Oxford Portable Electrospinner (OPE) [10] is a small, portable device that allows for a more flexible electrospinning (i.e., direction and type of target). Both the voltage (maximum voltage: 14 kV) and the polymer solution flow rate can be adjusted. The handheld apparatus has successfully created fibres from a range of polymers [14], including poly(vinyl butyral) (PVB), poly ε-caprolactone (PCL), and poly-(lactic-co-glycolic acid) (PLGA). The OPE has not been employed to produce nanoparticle-embedded fibres until now.

Biomedical applications of electrospun fibres have focused, mainly, on fibrous scaffolds for tissue engineering, wound dressing, antibacterial studies, biosensors, enzyme immobilization for faster reaction rates in biological reactions and drug delivery [4,5]. However, nanofibers have a high fabrication complexity and moderate biomimicry [15]. While the first issue is lessened with the use of a simple, portable electrospinning device, biomimicry can be increased with fibre modifications. Relevantly, a highly specialised ECM plays an instructive role in modulating cell behaviour, including the regulation of development, migration, function, and tissue repair [16]. By providing mechanical support and preferential attachment sites, an electrospun scaffold can guide cell proliferation (i.e., achieving directional growth) [2]. In brief, electrospun fibres can replicate some aspects of the native tissue and provide the relevant biochemical and physical cues at the appropriate times to create an optimal microenvironment and ensure regeneration.

Electrospun nano and microfibres may be natural, synthetic or hybrid (i.e., a combination of both) [7]. In this research, we opt for a combination of polycaprolactone and gelatine to create the electrospun fibres. As addressed by Unal [3], polycaprolactone is a synthetic polymer that offers advantages such as appropriate mechanical strength, biodegradability and non-toxic structural stability. Nevertheless, as a highly hydrophobic material, it is associated with decreased adhesion and reduced cell growth on its surface [3]. As such, it is necessary to combine it with another material in order to improve its surface chemistry and, especially, mimic certain topographic features of the ECM [16]. Gelatine, on the other hand, has excellent biocompatibility, is biodegradable, non-immunogenic and is an inexpensive material that may provide an additional 3D architecture for tissue engineering scaffolds [3]. As such, the combination of polycaprolactone and gelatine results in a bioartificial polymeric material with good biocompatibility, with improved mechanical and physicochemical properties. The biocompatibility of PCL-Ge electrospun scaffolds has been validated in vitro and in vivo in the literature [2,17].

Enhancing fibres with additional factors, such as those naturally present in the ECM (e.g., glycosaminoglycans, proteoglycans and glycoproteins), can help create an environment that is more similar to the natural tissue. The enhancement of electrospun fibres with antibiotics, analgesics and other drugs [9] and using them to create tissue engineering scaffolds is particularly attractive for site-specific delivery. Nevertheless, electrospinning drugs directly with the fibres might result in the loss of biological activity, low encapsulation efficiency, uneven drug distribution, compromised sterile environment, and burst release [4]. Moreover, the addition of drugs to the polymer solution could affect the polymer’s properties (e.g., viscosity), therefore impacting fibre fabrication [4].

Therefore, there is a need for methods that allow electrospun fibre functionalisation with, for instance, growth factors, antibiotics, antioxidants and drugs, without the limitations mentioned before. One of such methods encompasses nanoparticles, which can be loaded with several substances and then embedded into electrospun fibres. This strategy allows for a more versatile drug delivery system because both fibres and nanoparticles can be tailored to the specific needs of the tissue or patient. Drug-loaded nanoparticles and microspheres have been widely used for, among others, cancer [18]. Beyond loading nanoparticles with drugs, electrospun fibres can be functionalised with a variety of substances and biomolecules, yielding the opportunity to enhance performance and achieve additional functions [9].

Drug-loaded nanoparticle encapsulation, also considered to be a ”smart” drug delivery system [4], offers target-specific and triggerable drug delivery. NPs are usually embedded into electrospun fibres by direct incorporation during the electrospinning process or as a post-treatment.

In the first case, NPs are either added into the polymer solution and electrospun jointly, or electrosprayed directly onto the fibre surface during electrospinning [9]. In joint electrospinning, NPs are incorporated directly to the polymer solution before being loaded together into the electrospinning device. Alternatively, NPs can be incorporated before the electrospinning process by using a coaxial nozzle, where each of the two nozzles is loaded with either nanoparticles in a solution (usually, the core) or a polymer (commonly, the shell). This double layer overrides the sudden release associated with drugs electrospun jointly with the polymer solutions thanks to the barrier effect of the sheath structure [4]. This allows for multicomponent loading of drugs and their controlled released, which derives from the degradation of the shell layer [4].

On the other hand, post-electrospinning modifications protect bioactive agents from the electrospinning process itself, as well as permitting the addition of factors without the need to alter the electrospinning process or the polymer solutions. Nanoparticles can be generated on the surface of electrospun fibres by indirect fabrication through techniques such as (1) surface deposition, (2) in situ synthesis or (3) hydrothermal treatment [9,19]. The first method is the simplest one, and it comprises the immersion of the electrospun fibres into a colloidal suspension of NPs [20] with the aim of capturing these through hydrogen bonding, chemical binding [21] or electrostatic force [19]. Special care must be taken to ensure that the fibres are not soluble in the colloidal suspension containing the nanoparticles. Moreover, it is possible to add multiple layers of charged nanoparticles by opting for a layer-by-layer approach [19]. Nevertheless, a major drawback of this technique is that even distribution of NPs on the fibre’s surface is difficult to achieve.

In situ synthesis can improve distribution uniformity [9] and is achieved through a number of processes, including liquid-phase deposition, mainly used for metal oxide nanoparticles [22]; biomineralization, for calcium phosphate and apatite NPs [23]; and reduction or annealing of the absorbed precursor, which has been employed for metal and metal oxide nanoparticles [24]. Third, hydrothermal treatments have been employed to synthesise nanoparticles with different morphologies, determined by the hydrothermal conditions in place, like rods, plates, and spheres [9].

Direct incorporation of nanoparticles into the polymer solution prior to electrospinning allows for the positioning of NPs within the fibre instead of on the surface only and helps maintain a sterile environment. However, the electrospinning process may have an impact on the nanoparticles or on their release profile [9,25]. It is important to bear in mind not only the impact of the electrospinning process itself (e.g., mechanical stress), but also from the exposure to the solvent used in the polymer solution, which might be harmful to the NPs.

Post-electrospinning addition of NPs does not intervene with the electrospinning process and both burst release and short-term release are mitigated [25]. The two main limitations of this, however, are that achieving uniform distribution of nanoparticles along the fibres is challenging [9,25] and that an additional step after electrospinning is required. This could prevent direct fibre deposition onto, for instance, the wound site as enough time to ensure NP attachment to the fibre must be given before being able to place the scaffold in its final location.

For this research, chitosan (CS), a chitin-derived natural polysaccharide, and hyaluronic acid (HA), a non-sulphated glycosaminoglycan, were selected to create the nanoparticles. While hyaluronan is used in a variety of clinical applications [26] due to its non-immunogenic, mucoadhesive, analgesic and biodegradable properties [27,28], chitosan is biocompatible and biodegradable, and has high stability and low immunogenicity [26,29]. These have been used to fabricate tissue engineering scaffolds, which promote cell attachment, proliferation and viability [28]. Relevantly, chitosan can create nanostructures through electrostatic interactions, hydrophobic interactions, hydrogen bonds and van der Waals forces [29], and hyaluronic acid has the ability to target specific cells by binding with receptors on the cell surface such as CD44 [30,31], which makes it suitable for drug delivery targeted at tumours [29].

**Figure 1:**
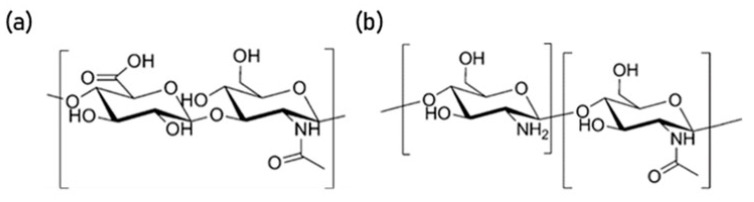
Molecular structure of (**a**) hyaluronic acid and (**b**) chitosan [34].

HA and CS nanoparticles have been synthesised by a number of methods, including complex coacervation [26] and ionic gelation [27,32,33]. Briefly, HA, a relatively high-molecular weight weak polyanion, and CS, a lower molecular weight weak polycation, create an asymmetric polyelectrolyte pair [28,34]. They bind together due to electrostatic interactions between the free amino groups in chitosan and the carboxyl group in hyaluronan [28] (Figure 1).

Ionic gelation (ion-induced gelation) is a simple process that requires the mixing of two aqueous phases at room temperature [32]. It involves an opposite charge ionic polymer (e.g., sodium triphosphate pentabasic—Na_5_O_10_P_3_) that initiates cross-linking. When dealing with polyanions or polycations, the electroneutrality principle cannot solely be accountable for the cross-linking; thus, additional elements (e.g., presence of other groups, 3D-structure) are considered to impact conjugation functionalities [32,33].

Controlled-sized nanostructures, such as HA-CS polyelectrolyte complex nanoparticles, are particularly useful for TE applications [29]. These nanosystems interact well with cell surfaces, which are negatively charged [28], and therefore offer prolonged residence time at the target site [28,35]. As reported by de la Fuente [27], systems incorporating HA have been used to modify surfaces with the aim of improving their adhesive properties, and have excellent capacity to associate either hydrophilic or hydrophobic macromolecules [29].

Nanoparticles offer high encapsulation efficiency and penetration ability, slow degradation rate, small mean size (10–1000 nm) and effective targetability [36]; characteristics that are particularly useful for biomedical applications. Clinical applications of HA-CS NPs include non-viral vectors for gene delivery [26], protein or drug delivery [33], tumour-targeted magnetic resonance imaging (MRI) contrast agents and macromolecule micro/nanocarriers [27] with controlled release, including heparin [35], interleukin (IL)-1β [37], DNA and RNA [38].

This research aims to develop a biocompatible scaffold to enhance neural cell attachment and viability, as well as favouring directional growth. Furthermore, the main objective of this paper is to demonstrate that hyaluronic acid-chitosan (HA-CS) nanoparticles can be electrospun together with polycaprolactone and gelatine fibres using a portable apparatus to create scaffolds for tissue repair.

## 2. Materials and Methods

### 2.1. Electrospinnability and Characterisation

#### 2.1.1. Preparation of Polymer Solutions

Polycaprolactone (PCL; Sigma-Aldrich, UK) and gelatine from porcine skin (Ge; Sigma-Aldrich, Saint Louis, MO, USA) were used to synthesise the polymer solutions listed in Table 2. PCL and Ge were each dissolved at 8, 10 and 12% *w*/*v* concentration in trifluoroethanol (TEF; Sigma-Aldrich, Saint Louis, MO, USA), and then combined in the following PCL solution:Ge solution ratios: 85:15, 70:30 and 50:50. Solutions were magnetically stirred (110 rpm) overnight at room temperature and visually examined to verify homogeneity prior to electrospinning.

#### 2.1.2. Electrospinning

Using a portable electrospinning device, designed and fabricated by the group [10], fibres were electrospun and collected using a metal plate (ground electrode) and an aluminium foil-covered cylinder, positioned at 15 cm from the needle’s tip. Polymer solutions were loaded into modified 3 mL syringes and fitted with a G19 needle. Flow rate was set to 1 mL/min and voltage varied between 9.82–10.56 kV, depending on the polymer solution. Humidity and temperature were measured at 51% and 20.1 °C, respectively.

#### 2.1.3. Fibre Characterisation

Fibres were electrospun for 15 s and imaged using SEM. From the four samples prepared, fibre diameters (6 per area, total n = 24) were obtained for each polymer from randomly selected areas.

### 2.2. Cell Culture

F11 cells (ATCC, Manassas, VA, USA), a somatic hybrid cell line made of embryonic rat dorsal root ganglion (DRG) cells and mouse neuroblastoma (N18TG-2) cells, were cultured in T-25 and T-75 cell culture flasks (Corning Incorporated-Life Sciences, Oneonta, NY, USA) with Dulbecco’s Modified Eagle’s Medium (Gibco Life Technologies, UK), supplemented with 10% *v*/*v* foetal bovine serum (Gibco Life Technologies, Brazil) and 1% *v*/*v* Penicillin/Streptomycin (Gibco Life Technologies, Grand Island, NY, USA). Cells were incubated and medium was changed every 72 hours. Cells were used for experimental work at 80% confluency.

A small amount of culture medium was used to suspend cells when doing cell seeding during the experimental work, so that cells have maximum chance of being in contact with the electrospun fibres. Once cells attach to the fibres, more culture medium is added to supply nutrients to the cells.

#### Live/Dead Assay

A live/dead viability kit (Molecular Probes Invitrogen, Eugene, OR, USA) was used following the manufacturer’s instructions. Briefly, 20 µL of a 2 mM EthD-1 solution and 5 µL of a 5 mM Calcein AM solution were added to 10 mL of Phosphate-Buffered Saline (PBS; Gibco Life Technologies, Bleiswijk, The Netherlands). This solution was added to the cells following media removal and incubated for 30 min at room temperature, away from the light. Calcein AM was used to stain live cells green, while dead cells were stained red using EthD-1.

A microplate reader (SpectraMax i3x, Molecular Devices, San Jose, CA, USA) was used to measure the fluorescence of Calcein AM (530 nm, excited at 645 nm) and EthD-1 (485 nm, excited at 530 nm), separately, for each sample. Background fluorescence was subtracted from the readings prior to the calculation of the live cell percentage (Equation (1)).

(1) $$\%\mathrm{Live}\;\mathrm{Cells}=\frac{F{\left(530\right)}_{\mathrm{sample}}-F{\left(530\right)}_{\min}}{F{\left(530\right)}_{\max}-F{\left(530\right)}_{\min}}\times 100$$

### 2.3. Cell–Fibre Interaction

Fibres were electrospun for 60 s and sterilised by performing one wash with 70% ethanol, followed by three washes with PBS, and placing the samples under UV light (20 min). An aluminium foil-covered press was used overnight prior to fibre sterilisation to ensure that the fibres remained at the bottom of the plate so that they would be submerged in the culture media later on. Cells were seeded (300,000 cells/well) on top of these fibres (3 samples of each polymer) and incubated for 30 min in order to ensure cell attachment. Triplicates of the experimental and control (cells only) wells (48-well plate) were prepared.

Half a millilitre of additional fresh media was added, and cells were cultured for 72 h, after which average viability (Equation (2)) was determined using a live/dead kit. Based on the value exhibited by the control, some polymer fibres were selected for further analysis. This preliminary polymer selection was further refined based on cell density (Equation (3)), bearing in mind the relevance of cell attachment points.

(2) $$\mathrm{Cell}\;\mathrm{Viability}\;\left(\%\right)=\frac{\mathrm{Live}\;\mathrm{Cell}\;\mathrm{Acount}}{\mathrm{Live}\;\mathrm{Cell}\;\mathrm{Acount}+\mathrm{Dead}\;\mathrm{Cell}\;\mathrm{Acount}}\times 100$$

(3) $$\mathrm{Cell}\;\mathrm{Density}\;\left(\frac{\mathrm{cell}}{{\mathrm{mm}}^{2}}\right)=\frac{\mathrm{Average}\;\mathrm{Total}\;\mathrm{Cell}\;\mathrm{Acount}}{\mathrm{Area}}$$

### 2.4. Scaffolds

**Figure 2:**
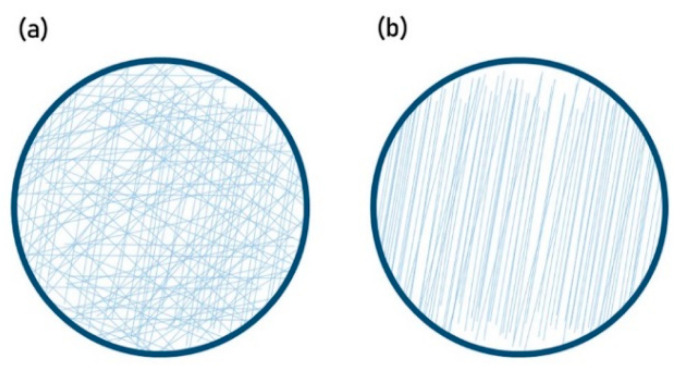
(**a**) Random and (**b**) unidirectional fibre arrangements as a schematic.

The selected polymers were used to create scaffolds in two different arrangements: random and unidirectional (Figure 2). Fibres were electrospun for 15 s to prepare each of five samples, and imaged using SEM. Fibre alignment was verified by quantifying angle variation (6 per area, total n = 30) of the unidirectional arrangements, measured via a reference angle (i.e., a horizontal line), using NIS-Elements AR. A 3° angle variation was deemed acceptable.

In order to evaluate cell interaction with the scaffolds, fibres were electrospun for 180 s and triplicates were placed in a 24-well plate. An aluminium foil-covered press was used overnight and, after removal, fibres were sterilised as outlined previously. Cells were seeded on top of the electrospun fibres (50,000 cells per well) and incubated for 30 min to ensure cell attachment. Then, they were cultured with additional 0.5 mL of media for 48 h. A control of cells-only wells was used, and triplicates were prepared. Viability was determined using a live/dead assay.

### 2.5. Fibre Functionalisation

#### 2.5.1. Hyaluronic Acid-Chitosan Nanoparticles (HA-CS NPs)

Hyaluronic acid (HA; Sigma-Aldrich, Prague, Czech Republic) and chitosan (CS; Sigma-Aldrich Life Science, Reykjavík, Iceland) were used to synthesise nanoparticles based on the method utilised by de la Fuente [27]. Briefly, a 1% *w*/*v* chitosan solution was prepared in a 10% aqueous solution of citric acid (Sigma-Aldrich, Budapest, Hungary), which was filtered and purified by dialysis in ultrapure water for 72 h. The volume was centrifuged (Heraeus Labofuge 400R, ThermoFisher Scientific, Willow Springs, NC, USA) for 1 h, at 25 °C and 4500 rpm before freeze-drying at −30 °C and 760 Torr with a VirTis AdvantagePlus (SP Industries Inc., Warminster, PA, USA). The resulting powder was dissolved (2.5 mg/mL) in an acidic medium (0.3% citric acid in ultrapure water), obtaining an aqueous solution. Separately, HA was dissolved in ultrapure water (0.75 mg/mL) and sodium triphosphate pentabasic (TPP; Sigma-Aldrich, Darmstadt, Germany) was incorporated (0.75 mg/mL). The volume was then filtered (0.22 µm filter).

Thirteen millilitres of the CS-solution were added to 6.5 mL of the HA-TPP solution and magnetically stirred (room temperature, 30 min), effectively synthesising the nanoparticles. The resulting sample was then centrifuged (1 h, 25 °C, 4500 rpm) to isolate the NPs. The remaining sample, in citric acid (0.3%), was filtered (0.45 µm filter) before use. When not in use, samples were stored at 4 °C.

#### 2.5.2. Characterisation

Size distribution was determined with Dynamic Light Scattering (DLS) using a Zetasizer Nanoseries Nano-ZS (Malvern Panalytical, Malvern, UK) at 23 °C. Briefly, a sample of nanoparticles dissolved in citric acid (0.3%) was analysed for three sessions of 90 runs of 90 s each. SEM analysis was performed on a 10 µL sample.

#### 2.5.3. Production Yield

Following centrifugation and filtration, supernatants were removed while the sediments were freeze-dried and weighted. Using the data gathered, production yield (PY) was obtained as Equation (4):

(4) $$\mathrm{Production}\;\mathrm{Yield}=\frac{\mathrm{Nanoparticle}\;\mathrm{Weight}}{\mathrm{Total}\;\mathrm{Weight}\;\mathrm{of}\;\mathrm{Solids}\;\left(\mathrm{HA}+\mathrm{CS}+\mathrm{TPP}\right)}\times 100$$

#### 2.5.4. Cytotoxicity

Cells were seeded in a 96-well plate, at a seeding count of 10,000 cells, and cultured with additional 200 µL of media for 24 h. Then, different concentrations (Table 3) of NPs were added, as well as a positive (ethanol 70%) and a negative (just cells; C0) control to be co-cultured for 48 h. Each concentration was tested five times.

Media was discarded and a solution of 100 µL of PBS and 20 µL of MTS reagent (Promega Corporation, Madison, WI, USA) was added to each well, which was then incubated for 60 min (37 °C, 5% CO_2_). Using a microplate reader, readings were taken every 10 min at 490 nm absorbance. Background fluorescence was subtracted prior to data analysis.

As the absorbance is proportional to cell density, cells-only wells were counted following standard cell culture procedure. The average cell count of the cell-only wells was equated to the average absorbance yielded. From here, associated cell count was extrapolated for the varying nanoparticle concentrations and, based on these, nanoparticle dosages were classified.

With the aim of identifying optimal and toxic doses, NP concentrations were classified as (1) toxic, those that were associated with a cell count lower than the initial seeding (10,000 cells/well); (2) non-toxic, where cell count was higher than the initial seeding but lower than the one exhibited by the cell-only wells; and (3) beneficial, which resulted in a cell count higher than both the initial seeding and the cell-only control.

### 2.6. Functionalised Electrospun Fibres

**Figure 3:**
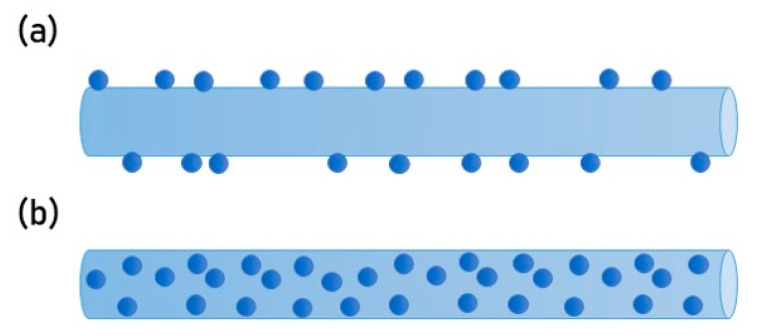
Schematic of fibre functionalisation with HA-CS nanoparticles as (**a**) surface modification or (**b**) blend.

PCL-gelatine fibres were electrospun for 300 s, as previously described, and functionalised with HA-CS nanoparticles (Figure 3), either attached to the surface (i.e., coating) or blended within the fibres. Fibres were arranged based on the results outlined by previous sections.

#### 2.6.1. Surface Modification

The PCL-Ge electrospun fibres were immersed into a 2 mL solution containing the HA-CS nanoparticles at the concentration deemed as beneficial (Section 2.5.4 and Section 3.4.3) and left overnight away from the light and inside a hood, allowing these to attach to the surface. The excess was carefully removed.

#### 2.6.2. Blend

The solution containing NPs at the optimal dosage was added to the polymer solution while undergoing magnetic stirring. Fibres were then electrospun as previously described.

#### 2.6.3. Cell Response

Surface-modified fibres and fibres containing NPs as a blend were electrospun separately for 300 s and placed in a 6-well plate. Fibres were secured to the bottom of the well and sterilised as previously described. Cells were seeded in each well (300,000) and co-cultured for 72 h, along with 1.5 mL of fresh media. Controls of unmodified fibres and cells only were used, and triplicates were prepared. Cell viability and proliferation were determined using a live/dead kit.

### 2.7. Imaging

A Nikon Widefield TiE2000 microscope was used to verify cell attachment before proceeding to incubation and monitoring of the samples. SEM imaging was performed using a Carl Zeiss Evo LS15 Variable Pressure (Germany) and images were analysed using its default software (ESEM). Samples were prepared for SEM imaging by placing them on an aluminium stub with a carbon adhesive. Then, they were gold-coated using a SC7620 Mini Sputter Coater System (Quorum Technologies, Ltd., UK).

### 2.8. Statistical Analysis

Where relevant, data was evaluated using one or two-way analysis of variance (ANOVA), with post hoc tests using Tukey’s honest significant difference (HSD), where *p* values ≤ 0.05 were deemed statistically significant. Data analysis was performed using Microsoft Excel 2019 (version 1908, Redmond, WA, USA) and presented as mean values with standard deviation (indicated by error bars).

## 3. Results

### 3.1. Electrospinnability and Characterisation

**Figure 4:**
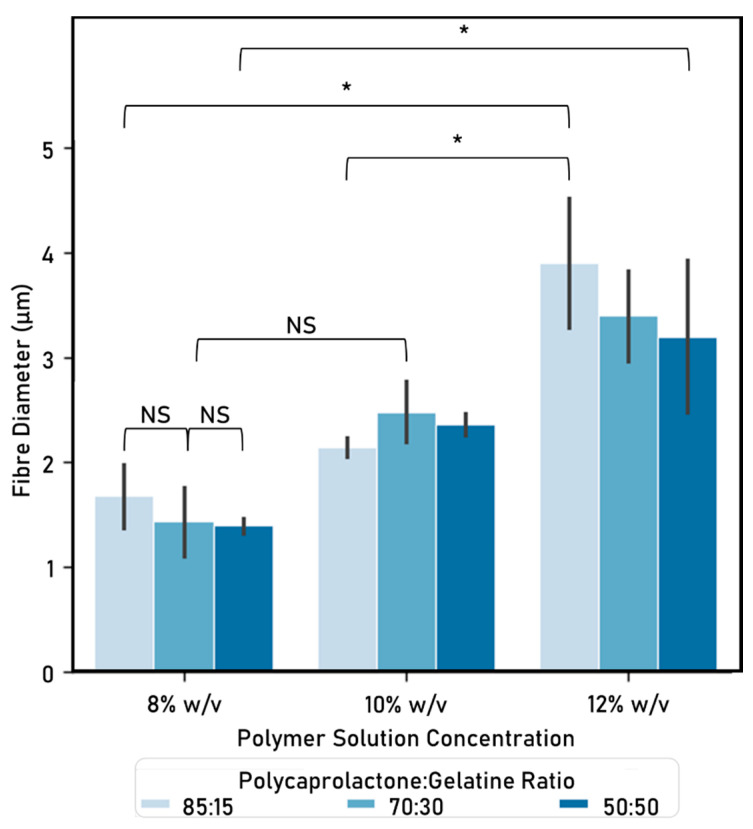
Fibre diameter (µm) (mean ± standard deviation). Electrospinning parameters: time: 15 s, solvent: TEF, voltage range: 9.82–10.56 kV, distance between nozzle and collector: 15 cm. Significant (*) and nonsignificant (NS) differences between samples are shown. (ANOVA/Tukey, *p* < 0.05; n = 24).

**Figure 5:**
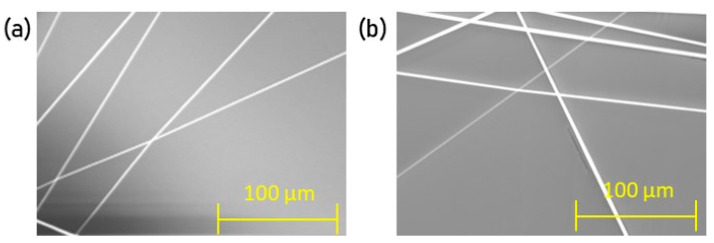
SEM imaging of electrospun fibres showing homogeneous morphology. Electrospun fibres from (**a**) 10% *w*/*v* at 70:30 polycaprolactone to gelatine solutions ratio and (**b**) 12% *w*/*v* at 50:50 PCL solution:Ge solution polymers shown. Electrospinning parameters: time: 15 s, solvent: TEF, voltage range: 9.82–10.56 kV, distance between nozzle and collector: 15 cm.

Electrospun fibres were successfully created using a portable device. All the polymers tested formed fibres, which were subsequently characterised in terms of diameter (Figure 4). The fibres produced have homogeneous morphology (Figure 5), as there is no presence of beads, curving or twisting.

As shown when comparing within the same PCL solution:Ge solution ratio, fibre diameters increase as the weight/volume concentration increases. The only case in which this did not happen, was when comparing 8% vs. 10% initial PCL or Ge in solvent within the 70:30 PCL solution:Ge solution ratio group. On the other hand, no statistically significant differences could be identified between PCL solution:Ge solution ratios within weight/volume concentration groups.

### 3.2. Cell–Fibre Interaction

**Figure 6:**
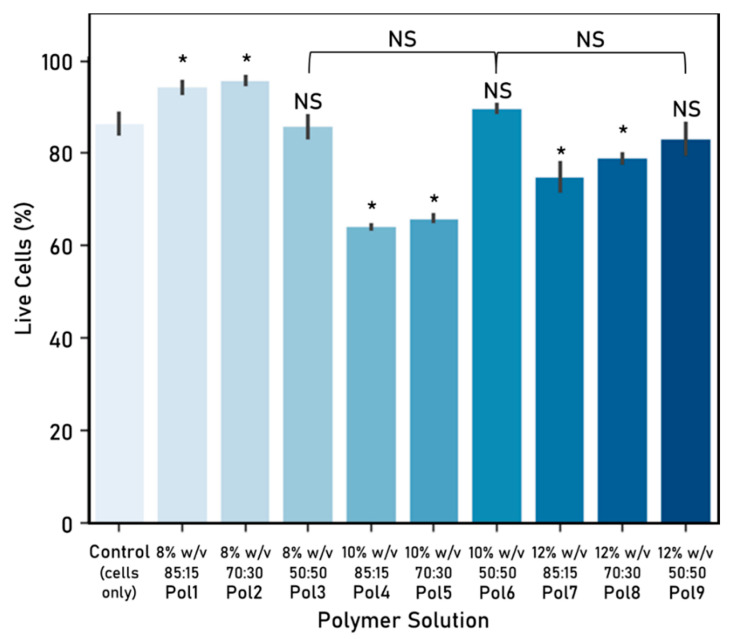
Cell–fibre interaction: cell viability (%) after 72 h culture (mean ± standard deviation). Cell seeding number: 30,000 cells, 48-well plate used, control: cells-only wells. Electrospinning parameters—time: 60 s, solvent: TEF, voltage range: 9.82–10.56 kV, distance between nozzle and collector: 15 cm. Significant (*) and nonsignificant (NS) differences between samples are shown. Unless otherwise specified, comparisons are made between the samples and the cells-only control. (ANOVA/Tukey, *p* < 0.05; n = 3).

**Figure 7:**
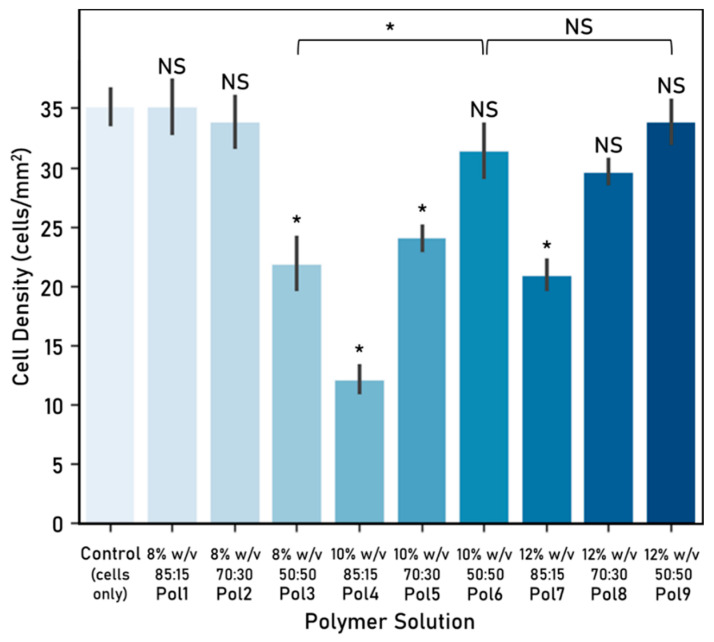
Cell-fibre interaction: cell density (cells/mm^2^) after 72 h culture (mean ± standard deviation). Cell seeding number: 30,000 cells, 48-well plate used, control: cells-only wells. Electrospinning parameters—time: 60 s, solvent: TEF, voltage range: 9.82–10.56 kV, distance between nozzle and collector: 15 cm. Significant (*) and nonsignificant (NS) differences between samples are shown. Unless otherwise specified, comparisons are made between the samples and the cells-only control. (ANOVA/Tukey, *p* < 0.05; n = 3).

Cellular response to the electrospun fibres was evaluated, in terms of cell viability (Figure 6) and density (Figure 7), and used to determine which polymer solutions are better suited to provide cell attachment points for directional cell guidance.

Polymers 1 and 2 were associated with a statistically significant higher viability in comparison with the cells-only control, while polymers 3, 6 and 9 were similar. Furthermore, the 50:50 PCL:Ge solution ratio behaved similarly across all weight/volume concentrations.

These polymers were further refined by looking at the associated cell density, which can be linked to the preference of cells to attach to fibres from that polymer. Polymers 3, 4, 5 and 7 were discarded as they yielded a statistically significant smaller cell density in comparison with the control. The other five polymers (Pol 1, Pol 2, Pol 6, Pol 8 and Pol 9) showed no statistically significant differences with the control and were therefore considered as suitable.

Considering both the cell viability and density, polymers 1, 2, 6 and 9 were considered for further study. Although there are no significant differences between them, polymer 6 was preferred over polymer 9 as it is associated with a higher cell viability. As such, polymers 1, 2 and 6 were selected for the further experimental work. It is worth highlighting that there seems to be no significant association between cell viability and density in the fibres tested.

### 3.3. Scaffolds

**Figure 8:**
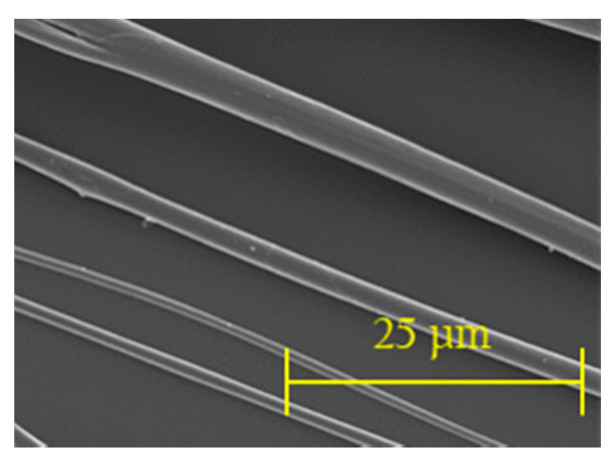
Unidirectional fibre arrangements (SEM, 300×).

The selected polymers (Pol 1, Pol 2 and Pol 6) were used to create two different fibre arrangements: random and unidirectional (Figure 8). The reliability of the unidirectional arrangement was corroborated by performing an alignment quantification (Table 4). As the variation was smaller than 3° across the tested polymers, the fibre arrangement was validated as homogeneous. No statistically significant differences between polymers exist, therefore confirming that the alignment is maintained across all polymers tested.

**Figure 9:**
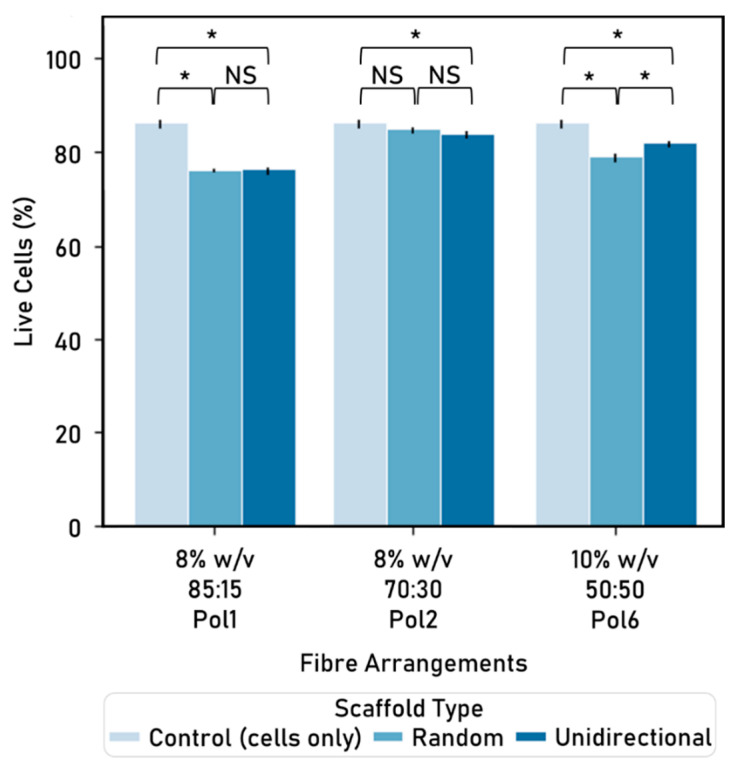
Cell viability (%) by polymer and fibre arrangement, after 48h culture (mean ± standard deviation). Cell seeding number: 50,000 cells, 24-well plate used, control: cells-only wells. Electrospinning parameters—time: 180 s, solvent: TEF, voltage range: 9.90–10.30 kV, distance between nozzle and collector: 15 cm. Significant (*) and nonsignificant (NS) differences between samples are shown. For comparison purposes, the control is repeated in every group. (ANOVA/Tukey, *p* < 0.05; n = 3).

Following a 48-h culture period, a live/dead assay was used to determine cell viability for these scaffolds (Figure 9).

Cell viability varies within arrangements: for instance, within the unidirectional arrangement, the 8% initial PCL or Ge in solvent at 70:30 PCL solution:Ge solution ratio polymer yields higher percentage of live cells than both the 8% 85:15 and the 10% 50:50 polymers.

When comparing the two fibre arrangements within the same polymer, similar cell viability occurs: for instance, 85:15 and 70:30 PCL:Ge solution ratio within the 8% *w*/*v* of initial PCL or Ge in solvent concentration. The only exception is Pol 6, where the unidirectional alignment is associated with a higher cell viability than the random arrangement. On the other hand, differences between polymers within the same type of fibre arrangement are significant.

The random alignment of 8% initial PCL or Ge in solvent, 70 PCL solution:30 Ge solution is the only polymer/arrangement combination that is not statistically significant to the cells-only control. Furthermore, fibres made with 8% *w*/*v* at 70:30 PCL:Ge solution ratio yielded the highest percentage of live cells across all arrangements and were selected for further study. Moreover, there is no statistically significant difference between random and unidirectional fibre arrangements within this polymer.

### 3.4. Fibre Functionalisation

#### 3.4.1. Characterisation of Hyaluronic Acid-Chitosan Nanoparticles (HA-CS NPs)

**Figure 10:**
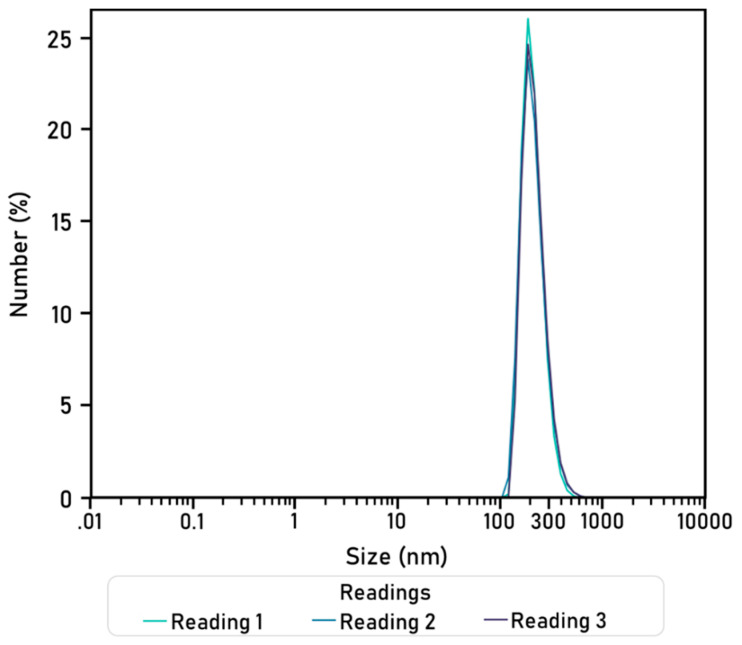
Hyaluronic acid-chitosan nanoparticle size (nm) distribution by number (%). Diameters were obtained in three readings (90 runs of 90 s).

**Figure 11:**
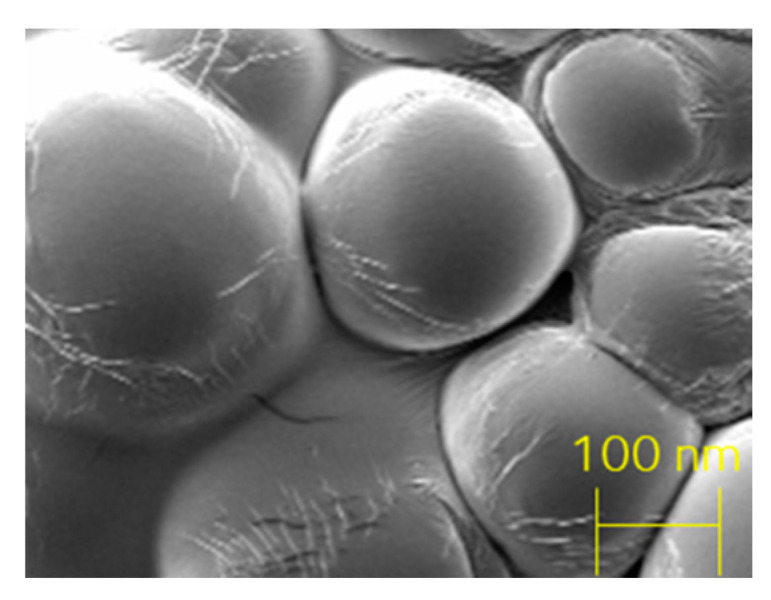
Hyaluronic acid (0.75 mg/mL)-chitosan (1% *w*/*v*) nanoparticles, SEM.

HA-CS nanoparticles were synthesised through ionic gelation and then characterised. Dynamic light scattering analysis (DLS) was performed thrice on a sample containing HA-CS nanoparticles dissolved in citric acid (0.3%). Readings were used to elaborate a size distribution graph by number (Figure 10) and, from here, obtain an average particle size and polydispersity index (Table 5). The synthesised HA-CS nanoparticles are spherical and have a smooth surface (Figure 11).

HA-CS nanoparticles were characterised with Dynamic Light Scattering, at 23 °C, with 3 readings of 90 runs (90 s each).

#### 3.4.2. Production Yield

Particle yield, calculated as the mass of dry HA-CS nanoparticles obtained per mass of the polymers used in the solutions at the start of the synthesis procedure, was 36.44%.

#### 3.4.3. Cytotoxicity

**Figure 12:**
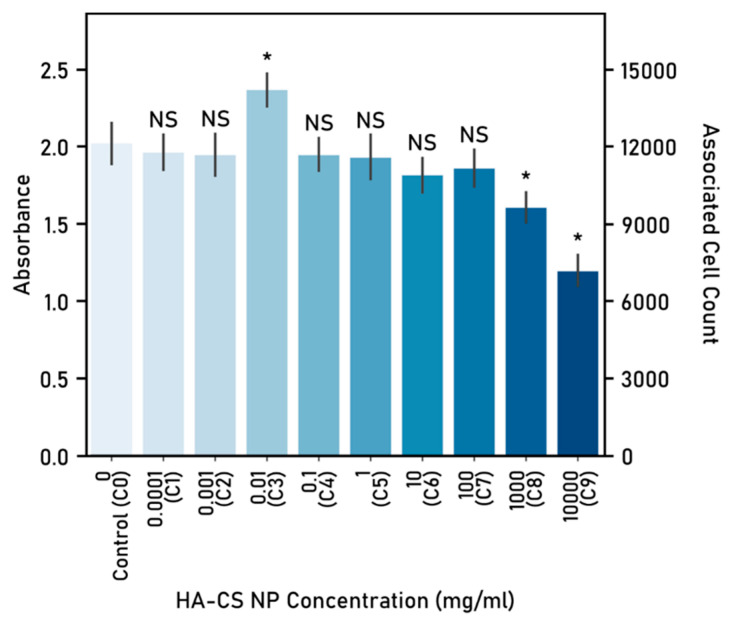
Determination of hyaluronic acid-chitosan nanoparticle dosage and associated cell count. Absorbance determined at 490 nm following 48 h of nanoparticle-cells co-culture. Cell seeding number: 10,000 cells, 96-well plate used, control (C0): cells-only well. Significant (*) and nonsignificant (NS) differences between samples and the control are shown. (ANOVA/Tukey, *p* < 0.05; n = 5).

Different concentrations (dosage) of HA-CS nanoparticles, as well as the cells-only control (C0), were tested to determine cytotoxicity (Figure 12). The average cell count of the cell-only wells was 1.2 × 10^4^ cells, which was used to extrapolate the associated cell count for the experimental dosages. Based on the associated cell count, nanoparticle dosages were classified (Table 6).

Two concentrations, 1 × 10^3^ mg/mL (C8) and 1 × 10^4^ mg/mL (C9), yielded a statistically significant lower cell count after co-culturing cells with the HA-CS nanoparticles. On the other hand, a single concentration (1 × 10^−2^ mg/mL) proved to be beneficial to cell proliferation, as it yielded an average cell count higher than the control. It was determined, consequently, as the optimal dose. The remaining six concentrations tested (C1, C2, C4, C5, C6 and C7) delivered a similar cell count to the cells-only control (C0).

### 3.5. Cell Response

**Figure 13:**
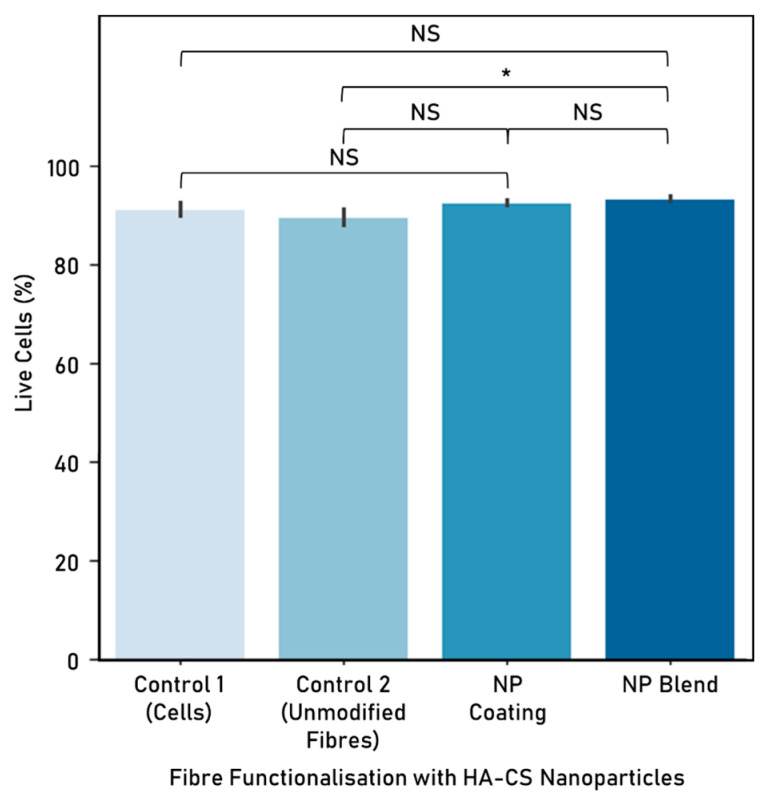
Cell viability (%) following 72 h co-culture with hyaluronic acid-chitosan nanoparticles (mean ± standard deviation). HA-CS nanoparticle concentration: 1 × 10^−2^ mg/mL. Cell seeding number: 300,000 cells, 6-well plate used, controls: cells-only (control 1) and unmodified fibres (control 2). Fibres were electrospun from a polycaprolactone-gelatine (70:30 PCL:Ge solution ratio) polymer, at 8% *w*/*v* initial PCL or Ge in solvent. Electrospinning parameters—time: 300 s, solvent: TEF, voltage: 10 kV, distance between nozzle and collector: 15 cm. Significant (*) and nonsignificant (NS) differences between samples are shown (ANOVA/Tukey, *p* < 0.05; n = 3).

**Figure 14:**
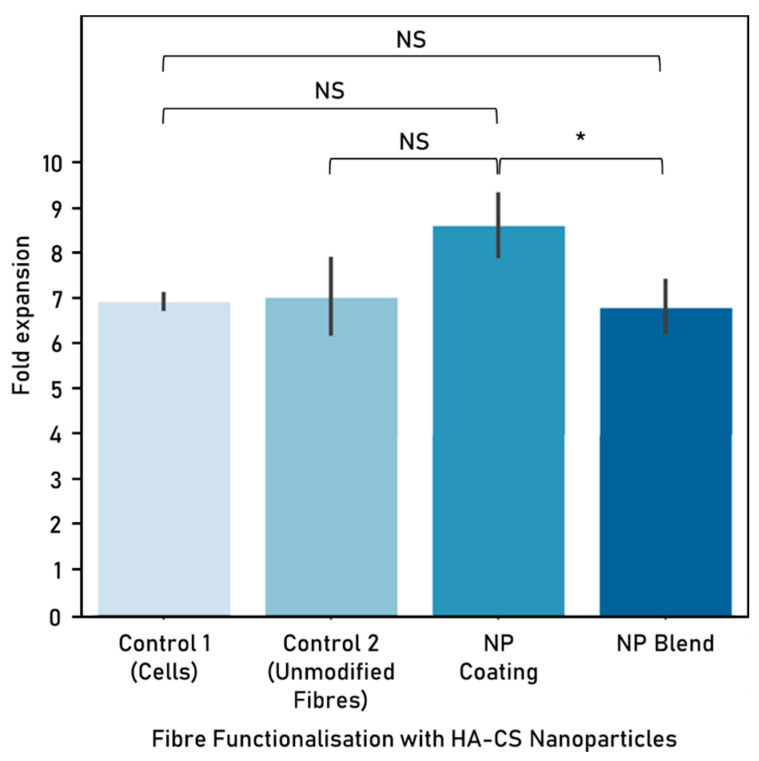
Cell proliferation (fold expansion) after 72 h co-culture with hyaluronic acid-chitosan nanoparticles (mean ± standard deviation). HA-CS nanoparticle concentration: 1 × 10^−2^ mg/mL. Cell seeding number: 300,000 cells, 6-well plate used, controls: cells-only (control 1) and unmodified fibres (control 2). Fibres were electrospun from a polycaprolactone-gelatine (70:30 PCL:Ge solution ratio) polymer, at 8% *w*/*v* initial PCL or Ge in solvent. Electrospinning parameters—time: 300 s, solvent: TEF, voltage: 10 kV, distance between nozzle and collector: 15 cm. Significant (*) and nonsignificant (NS) differences between samples are shown (ANOVA/Tukey, *p* < 0.05; n = 3).

HA-CS nanoparticles were used to functionalise electrospun polycaprolactone-gelatine fibres (8% *w*/*v* of initial PCL or Ge in solvent, 70:30 PCL:Ge solution ratio) either as a blend or a surface coating, at a concentration of 1 × 10^−2^ mg/mL. Nanoparticles were successfully integrated with electrospun fibres, which were then used to create a scaffold made up of unidirectionally-aligned fibres. However, NP distribution was not homogeneous and nanoparticle agglomeration sporadically occurred. Cell response to the PCL:Ge scaffolds embedded with hyaluronic acid-chitosan nanoparticles was evaluated based on cell viability (Figure 13) and proliferation (Figure 14), after 72 h of culture.

Embedding fibres with HA-CS nanoparticles, as a blend, resulted in higher cell viability in comparison with the unmodified PCL-gelatine fibres. In relation to the cells-only control, both techniques to embed NP to electrospun fibres resulted in similar cell viability.

In terms of cell proliferation, nanoparticles incorporated to the fibres as a coating performed better than when embedded as a blend.

## 4. Discussion

Developing scaffolds for tissue engineering that are both safe and able to guide cell proliferation is important for tissue repair. Electrospinning is a technique that has been employed for scaffold fabrication, particularly due to the benefits derived from the mechanical characteristics of the fibres it produces. Cells may fail to proliferate in a specific direction when a template is not provided, which is particularly common in in vitro testing and poses an additional challenge to successfully mimic the natural tissue. As such, an electrospun scaffold can guide cell proliferation by providing mechanical support and preferential attachment sites, as well as replicating to a certain extent some aspects of the native tissue. Cell proliferation guidance was achieved both in the unmodified fibres and the functionalised ones explored in this research.

A wide range of synthetic polymers, such as poly(vinyl butyral) (PVB), polydioxanone and polycaprolactone, can easily be electrospun into fibres by dissolving them in an organic solvent [2]. While they exhibit excellent mechanical properties, they lack cell-binding sites [3]. On the other hand, natural polymers, like gelatine and silk fibroin, have a good cell binding capacity and allow cell signalling and bioactivity [3], but face rapid or uncontrolled degradation rate and poor mechanical strength [7]. As such, combining these polymers into a hybrid, such as the case of the polycaprolactone-gelatine polymer explored in this research, seems to yield better results due to advantages like biocompatibility, maintaining differentiated function, provision of cell-binding sites and excellent mechanical properties [2,17], which are desirable elements for a tissue engineering scaffold.

In particular, the Oxford Portable Electrospinner has successfully electrospun a number of polymers, including the mixture of polycaprolactone-gelatine explored in this research. Fibres were successfully produced at different weight/volume concentrations (initial PCL or gelatine in solvent 8, 10, 12%) and PCL solution:gelatine solution ratios (85:15, 70:30, 50:50). 

The fibre diameters from the resulting fibres suggests that increasing the initial PCL or gelatine in solvent weight/volume concentration (e.g., from 8% to 10% at 85:15 PCL:Ge solutions ratio), results in a higher fibre diameter (increase of 1.672 µm to 2.138 µm). This observation is expected as weight concentration determines the viscosity and surface tension of the solution. Also, this affects the required electric field to form fibres, which alters fibre morphology [39]. Similarly, authors such as Chui [14], also report that fibre diameters increase when increasing the weight/volume concentrations.

This research implements a factorial design approach—varying the PCL solution to gelatine solution ratio, as well as the initial PCL/Ge in solvent—and was used to look at cell viability and proliferation. From here, further work is required to determine the effect of these variations and, furthermore, the optimal conditions for cell viability and proliferation considering biocompatibility, biostability and electrospinnability. This could be achieved by, among others, applying design of experiment (DOE) methods, using physical or statistical models [40]. DOE allows for a better understanding of the effects of different parameters, such as the ratio between PCL and gelatine, on the objective functions, including cell proliferation and viability. As such, it makes it possible to reduce the number of tests required, thus entailing minimal investment of time and resources, and is particularly used to optimise experimental formulations [40]. Conventional statistical experimental design helps determine the optimal conditions based on measured values of the characteristic properties [41], while other statistical design models, such as Taguchi, aim to identify optimal conditions by looking at the least variability [40,41]. In particular, formulation optimisation has been done using response surface methodology (RSM), where the mechanical and thermal properties of a polymer were optimised from different blend ratios [42]. Similar strategies could be implemented in further research.

Portable electrospinning devices grant a higher degree of flexibility and, as a result, make it possible to craft fibres in different arrangements as well as in situ fibre deposition onto the target site. Portable devices have a practical use in personalised advanced wound care, particularly when incorporating drug delivery applications, and tissue regeneration. Not only would it be possible to customise the scaffold to a specific site and patient, it could also take advantage of characteristic properties found in the natural tissue and minimise scaffold damage resulting from the process of implantation. Furthermore, multi-layered scaffolds (either of the same or different materials) could be placed directly in the target site. These would allow catering for a single scaffold for different types of tissue or with varying functionalisation depending on the layer.

Particularly, handheld devices have the potential to be used in a wide range of locations, including emergency medical transports, emergency settings and operating rooms. As such, this portability could simplify and accelerate the process of implanting a scaffold in vivo or achieving direct deposition of fibres functionalised with, for instance, nanoparticles loaded with antibacterial and antifungal agents onto wound sites. Moreover, these nanoparticles could be crafted so that they release their load at a later time, thus allowing for timely supply of, for example, antibiotics in burn wounds. Nevertheless, factors such as the duration of the battery, the toxicity of the solvent, and the amount of polymer solution that can be loaded in a cartridge must be taken into account at all times.

The cell viability experiments conducted in this research show that fibres do not provoke adverse effects on cell viability in the short term (up to 72 h); therefore, suggesting that polycaprolactone, gelatine and TFE (used here as a solvent) do not have a toxic effect that could lead to apoptosis. This is expected as other authors have created scaffolds from this combination of materials [17].

Also, cell viability does not seem to be significantly affected by the fibre arrangement. As such, scaffolds created with unidirectionally aligned or random fibres were associated with similar cell viability (e.g., 8% *w*/*v* initial PCL or Ge in solvent, 85:15 PCL:Ge solution ratio). In addition, unidirectional alignment offers the advantage of guiding cell proliferation in a uniaxial manner, which could be particularly interesting for nerve or muscle tissue repair. Although the unidirectional alignment (e.g., 84.26%, polymer: 8% *w*/*v* initial PCL or Ge in solvent 70:30 PCL:Ge solution ratio) yields a slightly smaller cell viability than the cells-only control (86.60%), the advantage of incorporating an electrospun scaffold is the capability to direct growth, which is particularly relevant for scaffolds intended to aid in injury repair and regeneration. Notably, when comparing different polymers (8% *w*/*v* initial PCL or Ge in solvent 85:15 PCL:Ge solution ratio, 8% *w*/*v* 70:30, and 10% *w*/*v* 50:50) within the same type of scaffold (unidirectional or random), there were significant differences based on cell viability. This suggests that polymer composition has a stronger influence on cell viability than fibre arrangements.

Some differences in cell viability can be identified when comparing the experimental work on cell–fibre interactions (Figure 6) with the one on fibre arrangements (Figure 9). These could potentially be attributed to the time cells were exposed to the electrospun fibres (48 h vs. 72 h).

The functionalisation of electrospun fibres is of particular interest to tissue engineering, as there is a need for nerve repair devices that mimic aspects of the native tissue and provide the appropriate biochemical and physical cues at the appropriate times. Replicating the natural environment more closely by, for instance, incorporating elements naturally present in the ECM can contribute to tissue repair and, if applicable, function restoration. Experimental work on modified fibres (i.e., coatings) with hyaluronic acid, chitosan, and NGF is included in the Supplementary Materials.

The second part of this research focuses on the functionalisation of fibres with nanoparticles. Hyaluronic acid-chitosan nanoparticles were successfully embedded into polycaprolactone-gelatine electrospun fibres using a portable apparatus to create scaffolds for tissue repair. This was achieved as either a direct blend of the NPs with the polymer solutions (i.e., prior to electrospinning) or a post-electrospinning modification.

While coatings allow fibres to be electrospun first and then modified, possibly making escalation more feasible, blending requires less steps, which can reduce the total scaffold production time. Embedding NPs as a blend enables the distribution of these throughout the electrospun fibres rather than on the surface only, as it occurs with the coating. This allows for a more uniform distribution along the fibres, but it is important to consider the impact of putting the NPs in direct contact with the organic solvent and the mechanical stress that NPs endure while going through the electrospinning process.

Post-electrospinning addition of nanoparticles (i.e., coating) was achieved by immersing the electrospun fibres into a colloidal suspension of HA-CS NPs. Pointedly, nanoparticles bind to the fibre surface by adsorption. Incorporating NPs as a post-electrospinning modification of fibres can prevent bioactive agents from destabilisation and denaturalisation during the electrospinning process. Moreover, the polymer solution’s properties, such as viscosity, are not impacted when adding the NPs.

In this research, we looked at the differences in cell viability and proliferation when comparing fibre functionalisation methods. When exposing these functionalised scaffolds to cells, incorporating NPs into fibres as a blend delivered a higher viability than the unmodified fibres control, while opting for a NP coating yielded higher cell proliferation. It is possible that embedding nanoparticles as a blend is not as successful as attaching the NPs to the surface because the area that is in contact with the cells is greatly reduced.

As both nanoparticle functionalisation options performed similarly to unmodified fibres in terms of cell proliferation, this demonstrates that incorporating NPs into fibres does not alter negatively cell response. Moreover, enhancing electrospun fibres with nanoparticles can allow for a number of clinical applications, including drug delivery. The controlled release of drugs carried within the NPs is not demonstrated in this research.

Nanoparticles are particularly interesting for biomedical applications, mainly because they offer high encapsulation efficiency, are able to penetrate tissues, usually have a slow degradation rate, have a small mean size, and effective targetability [36]. HA-CS nanoparticles have been used for a number of clinical applications, including protein and drug delivery [26], contrast agents and macromolecule carriers [27,37,38]. These nanoparticles are particularly useful for tissue engineering applications, as they interact well with cell surfaces and offer prolonged residence time at the target site [35]. Due to these advantages, this research is interested in incorporating nanoparticles into the tissue engineering scaffolds with the long-term aim of achieving drug deliver.

In particular, HA-CS NPs have the advantage of good biocompatibility, biodegradability, non-toxicity and non-immunogenicity, which make them ideal carriers for the therapeutic drug delivery. Hyaluronic acid is able to target specific cells by binding with CD44, a cell surface receptor [30,31]. This is particularly useful for tumour-targeted drug delivery. Similarly, these NPs could be further enhanced by incorporating functional layers that can easily bind to specific receptors.

The hyaluronic acid-chitosan NPs synthesised in this research via ionic gelation have a smooth surface, are spherical, and their diameters are around 200–300 nm. Similar findings, as well as for particle size and particle yield, are reported by a number of authors, namely, Raik [28], Pornpitchanarong [33], Zhou [26] and de la Fuente [27]. Relevantly, a higher TPP concentration leads to a smaller particle size as a result of a more compacted nanostructure derived from strong electrostatic interactions [27,31]. Moreover, particle size in this research is slightly smaller than the mean particle size reported by de la Fuente [27], on which the protocol used in this research was based on. This difference could potentially be attributed to the addition of a filtration step, which was added to obtain a more homogeneous population of nanoparticles by discarding, for instance, aggregated nanoparticles. This can be corroborated by the small polydispersity value, as small PDI figures are associated with highly monodisperse samples while larger figures obtained when there is a very broad size distribution. PDI values within the 0.1 to 0.4 range were considered acceptable, as they indicate that there is an adequate narrow size distribution of nanoparticles [43]. The HA-CS nanoparticles synthesised in this research have a PDI that falls within this range, therefore suggesting that there is homogeneity in the particle population. The final composition of the nanoparticles, in terms of percentage of hyaluronic acid and chitosan, is not analysed in this paper.

While the PCL-gelatine electrospun scaffolds embedded with HA-CS nanoparticles are not expected to provoke any significant adverse effects, there are some concerns associated with the safety of nanoparticles, in general, for human health and the environment. It is possible to minimise or significantly reduce the risk of harm throughout the synthesis, functionalisation, use and disposal of nanoparticles by incorporating a safe by design (SbD) approach [44]. This proactive approach aims to achieve this by integrating early a safety assessment of the materials and their interaction as early as possible in the development process. Strategies such as designing out the hazard in surface functionalisation, reducing release, and standardising production and characterisation methods have been used for this purpose [44].

The use of low-hazard biomaterials significantly contributes to the SbD approach, as such, opting for biocompatible materials like polycaprolactone, gelatine, hyaluronic acid and chitosan can help to reduce the associated risk. Moreover, conducting safety assessment tests, such as the MTS assay used to evaluate the cytotoxicity of HA-CS nanoparticles on F11 cells, aid in the validation of safe nanoparticles. In here, the toxic dosages were eliminated and, therefore, exposure to a potentially hazardous amount is prevented.

In this research, the nanoparticle synthesis method is based on electrostatic interactions between HA, CS and the cross-linker agent TPP. As it occurs in aqueous media, it avoids organic solvents, high temperatures and shear rates, all of which carry a risk of generating safety concerns. Moreover, ionotropic gelation as a synthesis method for HA-CS nanoparticles has been proven to result in low-toxicity nanoparticles [27].

UV light was used to sterilise the electrospun fibres before cell seeding. Exposure to UV light is a simple, low-cost and widely used method for fibre sterilisation. Relevantly, it does not influence fibre morphology or alignment, does not cause a critical effect in the physicochemical properties, and allows for cell adhesion and proliferation [45].

This study has a number of limitations. First of all, the maximum voltage that could be applied was 13 kV, due to the physical limits of the device’s converter. As applied voltage has an impact on fibre morphology [39], a wider range of voltage could have allowed us to achieve smaller fibre diameters. Another challenge derived from the use of the portable apparatus, which also affects fibre diameter is the solution feed rate [39]. Fibre production is limited by the amount of polymer that can fit into the syringe cartridge. Thus, long electrospinning sessions or those requiring a high polymer solution feed rate would require frequent cartridge replacements, therefore posing a problem in terms of scalability. These could be addressed by incorporating continuous feed of the polymers into the apparatus.

Moreover, the plates in which the experiments were carried out, do not mimic the natural tissue in both physical and biological terms. While well plates are readily available and allow for specific data collection, key physiological interactions are missing. Finally, as the experimental work covered in this research is based on the short-term (up to 72 h) response of the cells to both the fibres and nanoparticles, longer term performance of the scaffold, including degradation testing, ought to be explored.

Further work on nanoparticle loading, characterisation of the release profile of loaded NPs and an assessment of controlled release of these would allow continued development of these scaffolds for clinical applications. Additional experimental work is required in order to obtain homogeneous distribution of HA-CS nanoparticles along the fibres, which could potentially be achieved by incorporating a coaxial nozzle into the portable apparatus or adapting the cartridge to allow for continuous mixing.

## 5. Conclusions

A significant challenge to the successful mimicking of the natural tissue derives from cells failing to proliferate in a specific direction when a template is not provided, particularly common in in vitro settings. This research aims to develop a biocompatible scaffold to enhance cell attachment and viability, as well as favouring directional growth. Electrospinning is a remarkably simple and versatile technique that has been widely used for tissue repair and regeneration. With it, it is possible to achieve the desired structure and properties (e.g., porosity, diameter, alignment and biodegradability) by modifying different parameters, thus allowing for widely customisable scaffold fabrication. Electrospinning also makes it possible to generate scaffolds that mimic the hierarchical structure of the ECM, which are critical for cell attachment and proliferation.

In tissue engineering particularly, the relevance of artificial scaffolds that mimic the natural structures and exhibit similar biological properties is key for tissue repair and regeneration. Moreover, the performance of these scaffolds also depends on the cytocompatibility and affinity to the tissue, on top of the durability of the scaffold itself. Therefore, the degradation rate of these polycaprolactone-gelatine scaffolds embedded with hyaluronic acid-chitosan nanoparticles must be explored.

In addition to the topographical cues provided by the electrospun fibres, these can be complemented with electrochemical and biochemical cues thanks to the addition of loaded NPs. Moreover, the release of the substances contained within them could be designed for controlled release, upon the interaction with a specific substance or exposure to stimuli, or even based on the degradation of the NP shell. Not only does this hold a great potential for drug delivery, but also to induce wound healing by releasing factors that promote cell migration to the injured site and the liberation of anti-infection and anti-inflammation by carrying antibiotics or other drugs with antibacterial or antifungal properties, thus promoting effective repair.

Biocompatible scaffolds in different arrangements and from a range of polymer solutions were successfully created with a portable device. Furthermore, fibre modifications and functionalisation were explored as an approach to biomimicry. Relevantly, PCL-gelatine electrospun fibres, including functionalised with nanoparticles, allow cell attachment and promote directional growth.

Finally, the OPE successfully electrospun fibres embedded with nanoparticles as a blend, a previously unexplored variation for this device. This is particularly interesting because portable electrospinning devices are particularly useful for direct fibre deposition onto the damaged site, which allows it to be tailored to individual patients. This could minimise any risks of contamination post electrospinning and avoid adverse effects of sterilisation after fabrication. Moreover, it has potential as a drug delivery agent, as macromolecules encapsulated within the NPs could be released when implanted in the body, at specific time points, or when exposed to external stimuli (e.g., electromagnetic field and environmental cues).

## Figures and Tables

**Table 1:** Advantages and disadvantages of electrospinning apparatus: comparison between benchtop and portable devices. Adapted from Mouthuy, 2015 [7,10,12].

	Benchtop Devices	Portable Devices
Advantages	♦ Control over ambient parameters	♦ Portability (e.g., light, small and handheld)
	♦ Higher voltages available	♦ Flexibility in use (e.g., direction and target type)
	♦ Accuracy on voltage and flow rate	♦ In situ spraying and/or spinning
	♦ Safety management	♦ Battery or generator-powered
	♦ Can produce a large volume of fibres	♦ Affordable
Disadvantages	♦ Lack of flexibility (e.g., stationary design)	♦ Fixed cartridge size (limited production unless cartridge replacement or continuous material feed)
	♦ Need for power source	♦ Limited by performance of converter
	♦ Difficult to transport (e.g., heavy, bulky and large)	♦ Limited by battery capacity
	♦ Expensive	

**Table 2:** Polymer solutions to be prepared from polycaprolactone (PCL) and gelatine (Ge) in trifluoroethanol (TFE).

Polymer	PCL Solution:Ge Solution Ratio (*v*/*v*)	Initial PCL in Solvent (% *w*/*v*)	Initial Ge in Solvent (% *w*/*v*)
Pol 1	85:15	8	8
Pol 2	70:30	8	8
Pol 3	50:50	8	8
Pol 4	85:15	10	10
Pol 5	70:30	10	10
Pol 6	50:50	10	10
Pol 7	85:15	12	12
Pol 8	70:30	12	12
Pol 9	50:50	12	12

**Table 3:** Hyaluronic acid-chitosan nanoparticle (HA-CS NP) experimental dosages.

	NP Concentration (mg/mL)
C0	0
C1	1 × 10^−4^
C2	1 × 10^−3^
C3	1 × 10^−2^
C4	1 × 10^−1^
C5	1 × 10^0^
C6	1 × 10^1^
C7	1 × 10^2^
C8	1 × 10^3^
C9	1 × 10^4^

**Table 4:** Quantification of Unidirectional Fibre Arrangement (in °).

Initial Weight/Volume Concentration of PCL and Ge in Solvent	PCL Solution:Ge Solution Ratio (*v*/*v*)	Standard Deviation (in °)
8%	85:15	1.102
8%	70:30	1.342
10%	50:50	1.496

Polycaprolactone (PCL)-gelatine (Ge) fibres. Quantification of angle variation measured via reference angle. Acceptable variation: up to 3°. Electrospinning parameters—time: 15 s, solvent: TEF, voltage range: 9.90–10.30 kV, distance between nozzle and collector: 15 cm. (ANOVA, *p* > 0.05, n = 30).

**Table 5:** Hyaluronic acid-chitosan nanoparticle (HA-CS NP) characterisation: mean particle size and polydispersity index (PDI).

HA-CS NPs	Average
Size ± std. dev. (nm)	218.31 ± 60.21
PDI	0.357 ± 0.019

**Table 6:** Classification of HA-CS Nanoparticle Dosage based on Associated Cell Density.

	NP Concentration (mg/mL)	Associated Cell Density (cells/well)	Classification
C0	0	12,000	Control
C1	1 × 10^−4^	11,663	Non-toxic
C2	1 × 10^−3^	11,570	Non-toxic
C3	1 × 10^−2^	14,069	Beneficial
C4	1 × 10^−1^	11,575	Non-toxic
C5	1 × 10^0^	11,476	Non-toxic
C6	1 × 10^1^	10,779	Non-toxic
C7	1 × 10^2^	11,041	Non-toxic
C8	1 × 10^3^	9529	Toxic
C9	1 × 10^4^	7109	Toxic

Cytotoxicity of hyaluronic acid-chitosan nanoparticles (HA-CS NPs) was classified based on the associated cell count. A control (C0) of cells without nanoparticles was used to calculate the associated cell count for the experimental wells. Cell counts were classified as follows; “Toxic”, where the cell count is lower than the seeding count (10,000 cells); “Non-toxic”, cell count lower or equal to the control but higher than the seeding count; and “Beneficial”, cell count higher than C0.

## Supplementary Materials

The following are available online at https://www.mdpi.com/2079-4991/10/10/2016/s1, The impact of surface modifications of polycaprolactone-gelatine electrospun fibres on cell viability is available as supplementary material. Hyaluronic acid (0.1%), chitosan (1%) and nerve growth factor (50 ng/mL) coatings are evaluated and are available online at Supplementary materials as Figure S1: Cell viability (%) by coating, after 72 h culture.

## Abbreviations

The following abbreviations are used in this manuscript.

CS	chitosan
DLS	dynamic light scattering
DOE	design of experiments
ECM	extracellular matrix
Ge	gelatine
HA	hyaluronic acid
PBS	phosphate buffered saline
PCL	polycaprolactone
PDI	polydispersity index
MRI	magnetic resonance imaging
NGF	nerve growth factor
NP	nanoparticle
PY	production yield
RSM	response surface methodology
SbD	safety by design
SEM	scanning electron microscope
TE	tissue engineering
TEF	trifluoroethanol
