# Supplementary material for: Electrospun Fibres with Hyaluronic Acid-Chitosan Nanoparticles Produced by a Portable Device
Source: Nanomaterials (Basel). 2020 Oct 13;10(10):2016. doi: 10.3390/nano10102016 (PMC7601987; doi:10.3390/nano10102016)
Supplement: Supplementary file 1 [file nanomaterials-10-02016-s001.pdf]

nanomaterials

Supplementary Materials

Electrospun Fibres with Hyaluronic Acid-Chitosan Nanoparticles Produced by a Portable Device

Carla V. Fuenteslópez and Hua Ye *

Institute of Biomedical Engineering, Department of Engineering Science, University of Oxford, Oxford OX3 7DQ, United Kingdom;carla.fuenteslopez@eng.ox.ac.uk
* Correspondence: hua.ye@eng.ox.ac.uk

Supplementary Materials: Fibre Modifications

1. Materials and Methods

Fibres were electrospun for 60 s and carefully placed in a 24-well plate (Corning Incorporated – Life Sciences, Oneonta, NY, US) in a unidirectional arrangement (i.e., parallel fibres). A press was placed overnight to secure the fibres to the bottom and, after it was removed, fibres were sterilised.

Three fibre coatings were explored: hyaluronic acid (HA; 0.1%), chitosan (CS; 1%) and neural growth factor (NGF; 50 ng/mL). Half a millilitre of each coating solution was added to each well (already containing the PCL-Ge fibres) and left for 3 hours at room temperature in order to ensure full fibre coating. After the excess was removed, samples were washed with half a millilitre of Phosphate-Buffered Saline (PBS; Gibco Life Technologies, Bleiswijk, the Netherlands).

Cells were seeded (50,000 cells/well) on top of the fibres and co-cultured for 72 h with 0.5 mL of media. Three fibre coatings (HA, CS and NGF) as well as controls of uncoated fibres and cell-only samples were used (n = 3). Cell viability was determined with the aid of a live/dead assay and a microplate reader, as previously outlined.

1.1. Hyaluronic Acid (HA)

A 0.1% solution of HA (Sigma-Aldrich, Prague, Czech Republic) in double distilled water was dissolved by magnetic stirring at room temperature until achieving homogeneity.

1.2. Chitosan (CS)

Chitosan (Sigma-Aldrich Life Science, Reykjavík, Iceland) was dissolved at 1% in acetic acid (Sigma-Aldrich, Saint Louis, MO, US) by magnetic stirring at room temperature for 3 h. Then, three washes with a 0.1 M NaOH solution and deionised water were performed.

1.3. Nerve Growth Factor (NGF)

NGF (PeproTech, Cranbury, NJ, US) at a concentration of 50 ng/mL was dissolved in PBS and left to solubilise for 30 min, vortexing occasionally.

2. Results

Fibre modifications were achieved through coatings, which were evaluated using a live/dead assay. For each type of coating, as well as for the cell-only and unmodified fibres controls, cell viability was obtained (Figure S1).

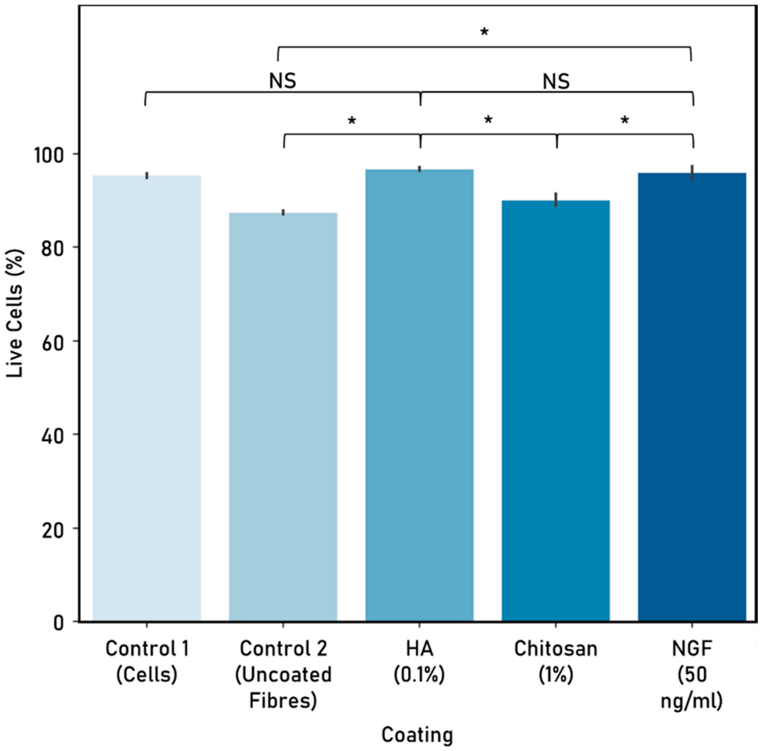

Figure S1. Cell viability (%) by coating (mean ± standard deviation), after 72 h culture. Cell seeding number: 50,000 cells, 24-well plate used, controls: cells-only (control 1) and uncoated fibres (control 2). Fibres were electrospun from a polycaprolactone-gelatine (70:30 PCL:Ge solution ratio) polymer, at 8% w/v initial PCL or Ge in solvent. Electrospinning parameters—time: 60 s, solvent: TEF, voltage: 10 kV, distance between nozzle and collector: 15 cm. Significant (*) and nonsignificant (NS) differences between samples are shown. (ANOVA/Tukey, $p < 0.05$; n = 3).

In comparison with the unmodified fibres control, both hyaluronic acid and NGF yielded a statistically significant higher viability. On the other hand, when comparing the experimental fibres with the cells-only control, only chitosan was associated with a statistically significant lower viability. The HA and NGF coatings performed similar to the cells-only wells; however, it is worth noting that the advantage of using either of the coated electrospun fibres relies on their ability to direct cell growth by providing preferential attachment points.

When comparing between coatings, both HA and NGF delivered a statistically significant higher viability than chitosan. This could potentially be attributed to the solution in which CS was dissolved in (acetic acid).

© 2020 by the authors. Submitted for possible open access publication under the terms and conditions of the Creative Commons Attribution (CC BY) license (http://creativecommons.org/licenses/by/4.0/).
